# An Initial Study of Ultra High Performance Concrete as Reusable Mold Material for Aluminum Casting

**DOI:** 10.3390/ma18010153

**Published:** 2025-01-02

**Authors:** Janna Link, Fabian Teichmann, Alexander Wetzel, Sebastian Müller, Bernhard Middendorf

**Affiliations:** 1 Department of Structural Materials and Construction Chemistry, University of Kassel, 34117 Kassel, Germany; 2 Institute of Casting Technology, Friedrich-Alexander-Universität Erlangen-Nürnberg, 90762 Fürth, Germany

**Keywords:** reusable mold casting, UHPC, surface characterization, aluminum, alkali-activated materials, die soldering

## Abstract

The initial investigation evaluates the feasibility of ultra high performance concrete (UHPC) as a material for reusable molds in aluminum casting. Two specific UHPC formulations were investigated: one based on ordinary Portland cement (OPC) and another utilizing alkali-activated materials (AAM). The study focused on investigating the surface through roughness measurements and the thermal durability through repeated casting cycles. The thermal stability of the molds was investigated by thermogravimetric analysis, mercury intrusion porosimetry, crack segmentation, optical microscopy, and electron microscopy. Results indicate that molds fabricated from AAM-UHPC exhibit relatively better performance in terms of maintaining structural integrity and surface quality over repeated uses. AAM-UHPC molds were able to withstand up to ten casting cycles with acceptable surface degradation and no significant failure, while OPC-UHPC molds exhibited a faster degradation under similar conditions. Microstructural changes and the interaction of UHPC materials with molten aluminum were investigated, highlighting the low adhesion and defect formation. Additionally, the molds demonstrated sound casting quality, with a grain size comparable to that achieved using traditional steel molds (~ 90 µm), underscoring the potential of UHPC materials for enhancing casting quality and efficiency. The study concludes that UHPC, particularly with alkali-activated formulations, shows promise for low-pressure casting environments.

## 1. Introduction

In the field of casting technology, processes exhibit a considerable degree of diversity, encompassing sand casting, which relies on single-use, expendable sand molds (commonly referred to as lost molds), and permanent mold casting, which employs reusable metal molds crafted from hot-work tool steels. Sand casting, which is distinguished by its versatile sand-binder systems, has a longstanding reputation due to its flexibility in producing complex geometries and accommodating small production runs at relatively low cost. Although sand casting is highly adaptable, it is inherently limited in terms of dimensional accuracy, surface quality, and the reusability of molds.

Recent developments have sought to address these limitations through the introduction of innovative sand-binder formulations and optimized gating systems [1]. In particular, additive manufacturing, notably binder jetting technology, has emerged as a transformative tool. This method enables the creation of intricate mold geometries directly from CAD models (Autodesk Inventor 2023), thereby eliminating the need for physical patterns or core boxes and significantly reducing lead time and labor costs [1,2]. Furthermore, the incorporation of enhanced shell-truss mold configurations through 3D printing has demonstrated the potential to improve thermal management, regulate cooling rates, and minimize prevalent defects such as porosity and inclusions [3].

However, the high energy consumption associated with traditional sand casting has given rise to environmental concerns. In response, Saxena et al. conducted a comparative analysis between conventional sand molds and 3D-printed molds, employing sustainability metrics to underscore the reduced waste and resource consumption achieved through the advancements in additive manufacturing. This signifies a promising shift towards more efficient, sustainable, and high-precision sand casting processes, which can effectively bridge the gap between cost-effectiveness and enhanced performance [4].

At the opposite end of the casting spectrum is permanent mold casting, which employs reusable molds. These molds require rapid cavity filling to ensure complete filling, especially for thin-walled and large components. Thermal stresses resulting from the uneven temperature distribution in the molds due to molten metal injection, product removal, and rapid cooling with coolants contribute significantly to die damage. The durability of die casting dies typically extends up to 100,000 shots; however, individual regions start to exhibit surface cracks after only 5000 to 25,000 shots [5,6]. These cracks generally form in the low-cycle fatigue range [7]. Early analytical methods by Hattel and Hansen [8] predicted thermal stresses in molds. Recent studies [9] highlighted that critical cracking often occurs in stress-concentrated areas such as fillet radii and holes. Choi et al. [10] developed an energy-based model predicting die fatigue life under thermal stress with high accuracy by combining computational fluid dynamics and finite element analysis. This model correlates well with actual fracture locations and fatigue life, proving to be valuable for estimating the durability of hot-work tool steels. Further research into the mechanisms of die wear has identified thermal fatigue, aluminization, and oxidation as key factors in steel cracking, often initiated by surface micro-cracking and exacerbated by oxidation [11]. Die soldering, influenced by differential concentrations of iron and aluminum, has been recognized as a major cause of surface deterioration. While release agents provide a temporary solution by creating a protective film, their single-cycle efficacy and impact on component properties, and as well as environmental and cost implications, limit their practicality [12,13]. Recent advances in high-pressure die casting (HPDC) have seen the introduction of comprehensive coatings on dies, significantly reducing the need for lubricants and extending the lifespan of steel molds [14]. However, these coatings do not completely prevent die soldering but decelerate it [6,15]. Han [16] explained that soldering occurs when molten aluminum penetrates the steel through coating defects, resulting in the detachment of the hard ceramic coatings applied by PVD.

The consideration of fully ceramic materials for permanent mold casting is a promising alternative, although their applications in this context remain largely unexplored. Mechanical stability under sudden thermal loading is a crucial consideration for these materials. Additionally, the direction of heat flow—categorized as ‘hot shock’ or ‘cold shock’—significantly influences material behavior and modeling. This distinction is particularly important in casting applications, where thermal shock from the molten metal (hot shock) predominates. Lu et al. [17] made a significant contribution to this field by analyzing temperature and stress profiles under various conditions in convective media, focusing on a spectrum of Biot numbers and fracture criteria. Their work indicates that hot and cold thermal shocks lead to markedly different patterns of crack initiation and propagation due to the distinct stress fields generated within the ceramic structure. Understanding these disparities is key to overcoming the challenges posed by different thermal shock conditions. Han et al. [18] performed thermal shock case studies on various ceramic samples under different scenarios (hot shock, cold shock, and ceramic coating under shock), highlighting the critical impact of temperature-dependent material properties on enhancing the thermal shock resistance of ceramics, especially at elevated temperatures. Recent research [19,20] has focused on crack propagation in ceramics under thermal shock conditions using numerical models. Ricardo et al. [19] employed the Coupled Criterion (CC) and Cohesive Zone Model (CZM) to predict surface cracking in ceramics during quenching, while Wang et al. [20] used a phase-field model to investigate how initial crack features, such as angle and length, influence crack evolution.

Recently developed compositions of ultra high performance concrete (UHPC) based on ordinary Portland cement (OPC) and alkali-activated materials (AAM) offer promising properties for applications in the field of pressure-assisted mold casting processes. UHPC is characterized by a compressive strength of 150–200 N/mm² and improved durability compared to ordinary concrete. These properties are achieved by optimizing the packing density through the addition of fines such as silica fume and quartz powder and by significantly reducing the water/binder ratio (w/b) [21].

In the cement hydration process, calcium silicate hydrate phases (C-S-H) are formed from the raw materials of the cement clinker. These phases contribute to the mechanical properties of the final product. The superior mechanical properties of UHPC enable the manufacturing of slim structures and lightweight construction while maintaining high strength and stability. However, the dense structure results in limited thermal stability: at elevated temperatures, the vapor pressure of the water released from the crystalline phases cannot escape and builds up until the construction fails explosively [22].

To increase the temperature resistance of UHPC and avoid spalling damage, the water vapor pressure in the material needs to be reduced. This is achieved by adding cellulose fibers into the concrete mix. The fibers form a branched spatial network in the hardened material and degrade under high thermal load. Wiemer et al. [23] and Scheffler et al. [22] have shown that channels formed by the thermal decomposition of organic fibers can act as outlets for the water vapor pressure without further damaging the binder matrix. The investigations conducted by Scheffler et al. [22] demonstrated the thermal stability of UHPC with added cellulose fibers up to a temperature of 500 °C. Further investigations indicated that no spalling occurred up to 1000 °C. These findings have been applied to the AAM-UHPC mix to produce a heat-resistant binder system without the use of OPC.

Ultra high performance concrete can also be formulated with the absence of ordinary Portland cement (OPC). It has been demonstrated that alkali-activated materials (AAM) can be used instead of OPC [24,25]. AAM consists of reactive aluminosilicate components, which are often secondary raw materials from industrial processes, and an alkaline activator, which can be an alkali hydroxide or alkali silicate [26,27]. Commonly used precursors include fly ash and ground granulated blast furnace slag. Silica fume and metakaolin can also be utilized. Due to the pozzolanic properties of the precursors, activation is necessary for them to react in the binder system. Depending on the chemical composition of the precursors, the resulting spatial networks can be amorphous to nanocrystalline and form a dense structure that contributes to the strength and durability. With the alkalis from the activator solution and the aluminum-rich precursors, N-A-S-(H) phases are formed, where “N” can be any alkali. In contrast to the formation of C-S-H phases in OPC-based systems, no water is incorporated into the crystalline structures of the reaction products of AAM. In the case of calcium-rich precursors such as ground blast furnace slag, C-A-S-H phases are also formed [27,28].

Due to the different reaction products of AAM compared to OPC-based materials, some advantages arise, such as reduced susceptibility to sulfate attack and chemical permeation [29]. Challenges in the application of AAM include high shrinkage [30]. Secondly, flow properties and water demand cannot be optimized with superplasticizers, as the highly alkaline environment hampers the application of common admixtures in AAM. The processability of AAM can be improved by adding silica fume to achieve low w/b ratios and, therefore, high strength [24]. The compressive strength of the AAM used here is comparable to that of OPC-UHPC, which is therefore referred to as AAM-UHPC. While OPC-UHPC consists of C-S-H phases that decompose at high temperatures, phase transformations and recrystallizations have been observed in AAM. Comparing these two different behaviors under thermal loading, the study by Fernandez-Jimenez et al. [31] and the investigation by Amer et al. [32] both pointed out that AAM-based concretes exhibit significantly better mechanical properties without the risk of explosive failure. These properties are advantageous for the application of AAM-UHPC as a casting mold. Additionally, since cellulose fibers have been shown to improve thermal stability, they are also used in AAM-UHPC.

Despite decades of research and industrial efforts to prevent die soldering and intermetallic phase formation, current solutions such as release agents and coatings remain insufficient for permanent molds.

The realization of fully ceramic permanent molds is also hindered by manufacturing constraints and cost considerations.

Hence, the current study shifts the focus from traditional steel molds to innovative concrete molds specifically designed for non-ferrous casting applications. These mineral molds possess significant potential, not only due to their easy and cost-efficient manufacturing but also because of their ability to positively influence the casting process and the quality of the final aluminum parts. The results of the current investigations pertain to the interactions between the casting process and the concrete-based molds, evaluating their performance and the resulting casting quality by using different microstructural methods.

## 2. Materials and Methods

### 2.1. Definition of the Process Chain

**Figure 1:**
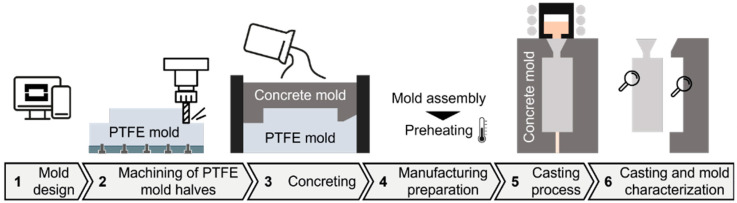
Schematic overview of the established process chain.

In order to achieve the objectives of this study, a comprehensive process chain was developed to facilitate the manufacture of UHPC molds and to enable casting trials to be carried out. The design and development of the process chain began with the creation of a CAD model, which was central to the design of the part and the creation of the initial casting mold model. This model was then inverted to produce a mold suitable for concrete casting. For simplicity, the chosen casting geometry consisted of a central sprue and two symmetrical risers. Following the design phase, the next step was to produce the concreting mold. This process entailed milling a block of PTFE that was secured to a stainless-steel plate to prevent any distortions during the milling process. Additionally, the potential of 3D printing for producing the concreting mold was investigated. However, challenges such as shrinkage, distortion, and poor surface quality led to a preference for milling over 3D printing, as illustrated in Figure 1. The milled PTFE molds were then used to cast two distinct formulations of ultra high performance concrete (UHPC), specifically ordinary Portland cement (OPC) and alkali-activated materials (AAM), as further detailed in Section 2.2. Following a curing period of 24 h, the concrete molds were demolded. Subsequently, these molds were assembled in pairs within a metallic frame and preheated to 300 °C at a rate of 600 °C per hour under ambient conditions using an N100 furnace manufactured by Nabertherm GmbH (Lilienthal, Germany). After preheating, the molds were transferred to the vacuum chamber of the VC-650V vacuum pressure-mold casting machine manufactured by BluePower Casting Systems GmbH (Walzbachtal, Germany). The chamber was evacuated before the molds were filled with molten aluminum at a maximum temperature of 720 °C and pressurized to 1.5 bar. This process facilitated solidification under pressure in an argon environment.

In order to evaluate the wear resistance of the molds, the concrete molds were filled with the aluminum alloy AlMg4.5Mn0.7, which contains 4.5% magnesium along with additions of manganese and chromium. This alloy is classified as a non-heat-treatable aluminum alloy, which is known for its exceptional strength characteristics. In its soft-annealed condition (O-temper), AlMg4.5Mn0.7 typically exhibits a yield strength (Rp0.2) of 150 MPa and a tensile strength (Rm) of 300 MPa. The alloy’s strength can be enhanced through cold working, and it also demonstrates excellent weldability and corrosion resistance, making it particularly suitable for use in seawater and general environmental conditions [29]. The observed enhancement in strength is primarily due to the solid solution strengthening facilitated by the magnesium content. Although AlMg4.5Mn0.7 is not commonly employed in conventional permanent mold casting processes, its utilization in non-metallic molds shows potential for attaining desired mechanical properties in the as-cast state. Notably, the content of entrained iron in the closely related AlMg5Mn0.8 alloy has been shown to play a significant role in the formation of Fe- and Mn-rich intermetallic phases [33]. Therefore, the alloy examined in this study is considered suitable for large-format vehicle structures and represents a viable alternative to the AlMg4Fe2 alloy (CastaDuct42), which is commonly employed in die casting applications [34].

### 2.2. Definition of the Concrete Materials

OPC-UHPC and AAM-UHPC were manufactured according to the research findings of Wetzel and Middendorf [24] and Wetzel et al. [25], which were based on the studies conducted by Scheffler [22] and Wiemer [23] on the use of cellulosic fibers.

In order to improve the thermal stability, cellulose fibers were admixed. To enhance the wetting capacity of the UHPC to the mold and thereby create a superior surface quality, a hydrophobic agent was employed (SikaControl AE-10, Sika AG, Baar, Switzerland). This also resulted in improved wetting and thus an improved surface in the experiments. Both AAM-UHPC and OPC-UHPC can be considered ultra high performance materials, exhibiting compressive strengths in excess of 150 MPa after 28 days. Cellulose fibers were added in an amount of 0.4 vol% to improve the thermal stability [22]. The components and masses for both mixes can be found in Table 1.

The concrete was manufactured using an Eirich 1 l intensive mixer (Gustav Eirich GmbH, Hardheim, Germany). Following mixing, the fresh material was filled into the pretreated molds while being vibrated at 50 Hz for two minutes. To facilitate demolding and minimize damage to the UHPC surface, the mold was pretreated with a PTFE release agent. The concrete molds were demolded 24 h after production [22,24].

### 2.3. Surface Characterization

The initial goal of this study is the introduction of UHPC molds. Therefore, the characterization of the surface of these molds is crucial to gain a further understanding of the wear over multiple casting cycles and the accuracy of the molds. As a result, the concrete molds were subjected to the following microscopic wear testing procedure: Using an Olympus OLS 5100 laser scanning microscope (Olympus K. K., Tokyo, Japan), 100 images and surface roughness measurements were taken on each mold used. The mold accuracy was quantified using two parameters: the arithmetical mean height (S_a_) and the developed interfacial area ratio (S_dr_). Sa represents the mean difference in height of each point from the arithmetical mean plane of the surface and is used to evaluate the roughness of the mold/casting surface in three dimensions. With the aim of quantifying the differences in surface roughness of the mold and casting surface after each step of the proposed manufacturing chain, S_dr_ was measured. The S_dr_ value expresses the percentage of the additional surface area contributed by the texture of the molds/castings surface topology. To achieve the above objective, the PTFE model, the mold, and the casting itself were measured at 100 points evenly distributed over the surface of interest. The casting mold was measured after eight castings, as were all the aluminum single castings. In addition to the surface measurements, the thickness of the castings was measured with a micrometer at five points on five castings. The resulting 50 measurements were then compared to the intended thickness of 6 mm.

To quantify the crack networks in the concrete molds, the same microscopic images were analyzed for the number of crack pixels detected in each image of a concrete mold before casting and after eight casting cycles. Segmentation of crack and non-crack pixels was performed using DeepCrack [35], a pre-trained neural network designed to segment cracks in images of concrete structures, proposed by Liu et al. The neural network was programmed in Python and trained on a dataset generated by the authors. After image segmentation, the percentage of crack pixels was calculated for each image taken and averaged for each mold.

### 2.4. Microstructural Analysis

#### 2.4.1. Concrete Molds

In order to facilitate the porosimetric and thermogravimetric analyses, cube samples of the binders, with dimensions of 5 cm on each edge, were fabricated. Three different thermal exposure conditions were investigated. The samples were stored under standard climatic conditions (20 °C, 65% relative humidity) for a period of seven days prior to undergoing a heat treatment at 400 °C and 700 °C, respectively. Two distinct heat treatments were employed to investigate the potential for phase transformations at different temperature levels, with a reference sample stored at room temperature for comparative purposes. The samples were placed in a preheated oven (400 °C and 700 °C) for one hour, after which they were allowed to cool down to room temperature. The objective of this procedure was to simulate short-term thermal exposure in a broader sense in order to gain insight into the thermal loads experienced in casting processes. X-ray powder diffraction (XRD) and thermogravimetric analyses (TGA) were conducted on samples devoid of aggregates for the purpose of performing phase analyses. XRD measurements were conducted using a Bruker AXS 4D instrument (Bruker corporation, Billerica, MA, USA). However, the reaction products of alkali-activated materials (AAM) comprise nanocrystalline phases that are not detectable by X-ray. In contrast to the distinct peaks observed in crystalline phases, an amorphous hump can be observed between 20 and 35° 2θ. To evaluate the thermal stability of the ultra high performance concrete (UHPC), TGA was conducted on samples devoid of aggregates. Approximately 30 mg of ground material was heated to 720 °C at a heating rate of 10 °C/min, and the weight change of the sample was recorded using a Netzsch STA 449 F5-Jupiter instrument (Netzsch B.V. & Co. KG, Selb, Germany). Mercury intrusion porosimetry (MIP) was employed over a range of 0.0018–140 µm to measure the pore size distribution of the UHPC mixtures. The instrument used for MIP was ‘Poremaster’, produced by Quantachrome (Anton Paar QuantaTec, Boynton Beach, FL, USA). Prior to measurements, the samples were stored at a temperature of 40 °C for 24 h to ensure gentle drying. For the investigation of the microstructure of the concrete molds after casting, polished sections were prepared. These sections were obtained from a cross-sectional area, thus enabling an analysis from the external surface of the concrete mold to the internal surface that came into contact with the aluminum. An environmental scanning electron microscope (ESEM) operating in low-vacuum mode (40 Pa) and using a large-field detector was employed for secondary electron imaging (SE).

#### 2.4.2. Aluminum Castings

**Figure 2:**
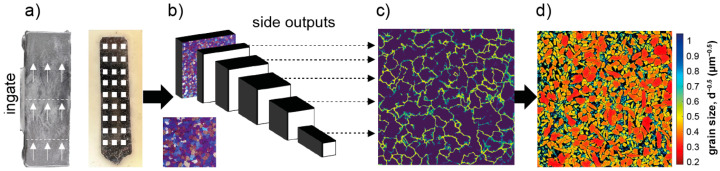
Schematic drawing of the course of the metallurgical examination: (**a**) extraction, (**b**) HED network, (**c**) edgemap, (**d**) grain size calculation.

In order to analyze the microstructure of the cast aluminum plates, three cross-sections were taken from two exemplary specimens (Figure 2), followed by grinding (P180, P320, P1200, P2500) and polishing (3 µm, 1 µm, 0.25 µm).

Subsequently, the cross sections were subjected to electrochemical etching using Barker’s acid at a voltage of 20 V and a current of I = 70 mA for t = 120 s. This method ensures that the grain boundaries are visible under polarized light, as illustrated in Figure 2. In particular, the grain boundaries are discernible in the images as a chromatic alteration, reflecting the variation in the orientation of the atomic grid of each grain. To extract the grain boundaries from these images, a pretrained neural network for holistically nested edge detection (HED) was applied [36,37] to each image taken. The network calculates a fused edge map from the side outputs of the network layers. The method for grain size determination in electron backscatter diffraction measurements from weldments, proposed by Lehto et al. [38], was adapted for use with the edge maps. This method allows the local grain size to be calculated from binary edge map images. All programming work was performed in MATLAB (version 2023b) and Python (version 3.6).

## 3. Results

### 3.1. Process Chain

**Figure 3:**
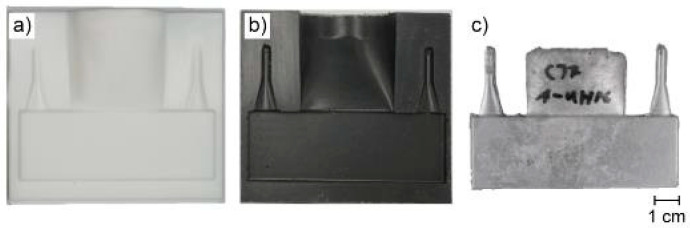
Overview of (**a**) PTFE mold, (**b**) AAM-UHPC-mold, and (**c**) resulting aluminum casting.

The process chain outlined in Section 2.1 yielded visually sound cast aluminum plates, as it can be derived from Figure 3. The PTFE mold (Figure 3a) was replicated in concrete (Figure 3b) and subsequently cast into an aluminum plate (Figure 3c). The casting trials demonstrated that the presented concrete molds are generally suitable for use as molding material. The experimental setup presented in Section 2 demonstrated that the OPC-UHPC molds were capable of surviving two casting cycles, while the AAM-UHPC exhibited durability of at least ten casting cycles. The failure of the molds was attributable to the formation of extensive crack networks within the concrete as a consequence of thermal and clamping stress.

The PTFE mold (Figure 3a) was manufactured by means of CNC milling. The manufacturing process and the material were selected in preference to additive manufacturing from polylactic acid or methacrylic acid, due to the surface quality and/or geometric distortion indicated by the initial tests. The results demonstrated that a smooth, nearly untextured surface of the mold is favorable to the production of concrete molds. Furthermore, the utilization of a hydrophobizing agent enhanced the wetting capacity by reducing the contact angle between the fresh UHPC and the PTFE mold. The resulting UHPC exhibited a smooth surface (Figure 3b) as well as a high dimensional accuracy, thereby demonstrating feasibility for the casting application. Similarly, the resulting aluminum casting also exhibited a homogeneous surface (Figure 3c), devoid of any voids or defects. At various points along the surface, minor elevations were observed. Moreover, in the region of the mold separation plane, aluminum was pushed out of the cavity, resulting in an unstable appearance of the edge of the castings.

The accuracy of the proposed casting process chain was evaluated on both macroscopic and microscopic levels. In consideration of the macroscopic measurements, the mean thickness of all castings tested was determined to be t = 5.96 mm ± 0.02 (UHPC and AAM-UHPC mold). A comparison of the thickness of the parts cast in AAM-UHPC and OPC-UHPC molds revealed a difference of 0.02 mm. It can therefore be concluded that the overall discrepancy between the intended and resulting thickness can be attributed to the differing accuracy of the used concrete molds. The resulting discrepancy between the mold materials were deemed to be insignificant, and the macroscopic accuracy of the entire process chain was calculated to be 99.33% from CAD to casting. It should be noted that the aforementioned accuracy assessment takes into account tolerances and shape accuracy, and that the entire process chain, which consists of two primary shaping processes, is regarded here.

### 3.2. Surface Quality and Wear of Concrete Casting Molds

#### 3.2.1. Surface Defects and Appearance

During the examination, all molds showed various cracks, networks of cracks, pores, and arrays of pores, which is not unusual for concrete materials and is associated with characteristic hardening processes such as shrinkage. The specific amount of cracks and pores, as well as their appearance, was dependent on the sort of concrete used. The surfaces of OPC-UHPC showed a smooth, flat surface with a few microscopic cracks unevenly distributed over the entire mold’s surface. After one casting cycle, significantly more and wider cracks in the OPC-UHPC surface were observed, and the number of thin cracks observed decreased vastly. Thus, it is presumed that the microcracks, which were present before casting, increased in width and length due to the exposition to mechanical and thermal stress during the casting process. The pores detected before casting were neither influenced in shape nor filled with aluminum. The AAM-UHPC surface appears rougher than the OPC-UHPC surfaces, exhibiting numerous macroscopic and microscopic cracks, along with crack networks homogeneously distributed across the entire surface. Despite the pronounced difference in surface characteristics, the AAM-UHPC molds were more resistant to the thermo-mechanical load resulting from the casting process. This behavior appears to be counterintuitive, but it is in fact a consequence of the way in which these crack networks of AAM function. The superior thermal behavior of AAM-UHPC indicates that the crack networks facilitate the escape of water vapor pressure. Previous studies have demonstrated that although shrinkage cracking occurs in AAM, the mechanical properties, such as compression strength, remain stable.

#### 3.2.2. Process and Mold Accuracy

**Figure 4:**
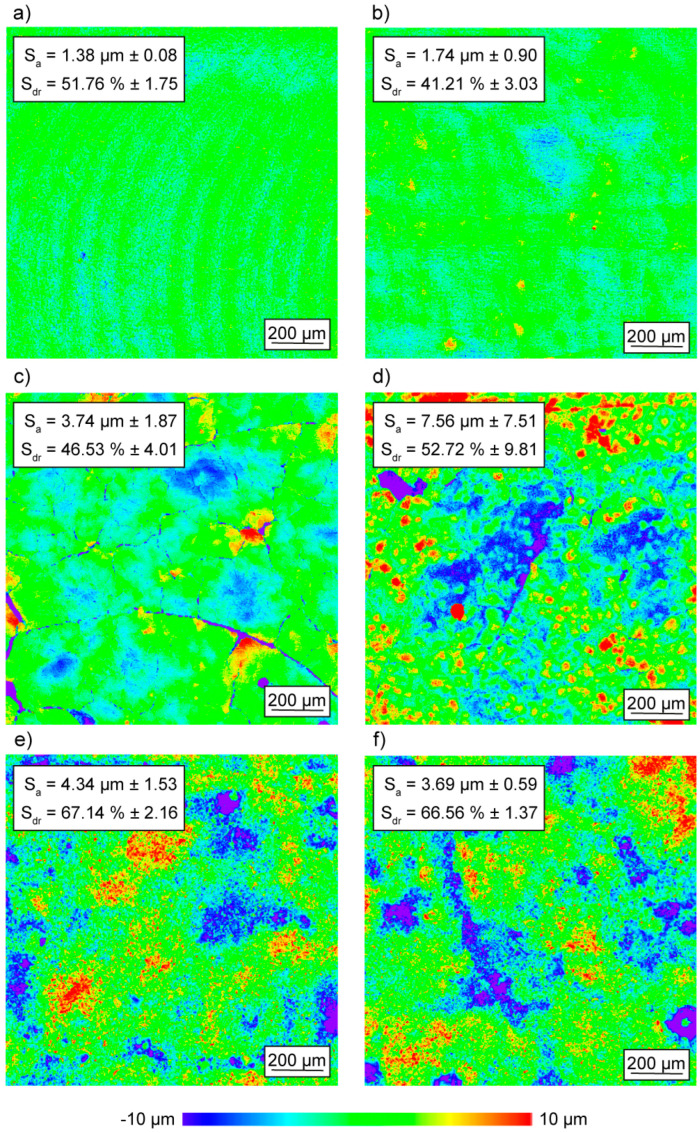
Representative surface topologies parameters refer to all measurements taken after n casting cycles: (**a**) PTFE model, (**b**) OPC-UHPC mold n = 0, (**c**) AAM-UHPC n = 0, (**d**) AAM-UHPC n = 8, (**e**) Al-Casting in AAM-UHPC n = 1, (**f**) Al-Casting in AAM-UHPC n = 8.

On the microscopic scale, the mold surface quality was measured to gain further understanding of the influence of the aluminum melt on the concrete molds in terms of microscale topology. Figure 4 presents representative results of the measurements taken by the use of confocal laser scanning microscopy. The figure displays the microscopic topologies of the surfaces of the PTFE mold (a), the OPC-UHPC mold before casting (b), the AAM-UHPC mold before (c) and after casting (d), as well as the aluminum part after one (e) and eight (f) casting cycles. Moreover, the surface parameters S_a_ and S_dr_ referring to all measurements taken are displayed. S_a_ defines the absolute value of the height difference in comparison to the average height of the whole area. S_dr_ defines the additional area that is generated by texture in comparison to a completely plain area. Both parameters combined give information about the texture and roughness of a surface.

Comparing Figure 2a–c implies that the average surface roughness S_a_ of the PTFE mold is sufficient to achieve sound concrete surfaces in both OPC-UHPC and AAM-UHPC, even though the PTFE model contained a periodical surface texture from the milling process (Figure 4a). Hereby, the average surface roughness slightly rises by ~0.4 µm in OPC-UHPC and ~2.4 µm in AAM-UHPC. Thus, the surface topology is more dependent on the concrete formulation than on the surface topology of the PTFE model. In addition, the texture of the milled PTFE model is obviously not transferred to the surface structure of both UHPC formulations, which is undermined by the drop in the S_dr_ value between PTFE and concrete. Before casting, the AAM-UHPC (Figure 2c) showed significantly more visual cracks than the OPC-UHPC concrete. Nevertheless, the OPC-UHPC molds failed after two casting cycles. After eight casting cycles of the AAM-UHPC molds, the cracks are less visible, and the average surface roughness doubles from S_a_ = 3.74 µm ± 1.87 to S_a_ = 7.56 µm ± 7.51, and the cracks become less visible. Moreover, the percentage of surface area contribution due to the texture (S_dr_) rises from 46.53% ± 4.01 to 52.72% ± 9.81. Presumably, this development is caused by the growth of the initial cracks detected in AAM-UHPC in the initial state, which leads to the detachment of single parts of the surface.

Comparing the surfaces of the aluminum castings, Figure 4e,f, the changes of the mold surface in roughness and texture are not transferred to the casting. The roughness of the mold surface (AAM-UHPC) increases significantly from S_a_ = 3.74 µm ± 1.87 before casting to S_a_ = 7.56 µm ± 7.51 after eight casting cycles, while the roughness of the casting steadily ranges around S_a_ ~ 4 µm.

Due to the fact that the AAM-UHPC molds (n_max_ = 8) exhibited much stronger initial cracking than the OPC-UHPC molds (n_max_ = 2), it can be derived that the initial visual cracks and crack networks are not the cause of failure for concrete casting molds. Aside from that, a general finding from the comparison of the surfaces shown in Figure 4 is that the PTFE model surface was smooth and periodically textured, the AAM-UHPC concrete surface was cracked and had higher roughness than the PTFE, while the aluminum castings were more even compared to the AAM-UHPC surfaces. Potential causes for the deviation of concrete and PTFE surfaces, considering the transfer of the texture, are shrinkage, wetting behavior, and various reactions during the hardening of the concrete. Later, during casting, it is assumed that the aluminum melt is not able to infiltrate the surface of the concrete, due to the surface tension of the aluminum and the wetting behavior of the concrete surface. This was even observed when the surface became rougher due to the surface wear after eight casting cycles; see Figure 2c,d. It has to be taken into account here that all these observations are a function of the pressure in the cavity, as known from other casting processes such as high-pressure die casting, and were achieved under the circumstances described in Section 2. Changing the casting process or the mold geometry can therefore result in a different outcome.

**Figure 5:**
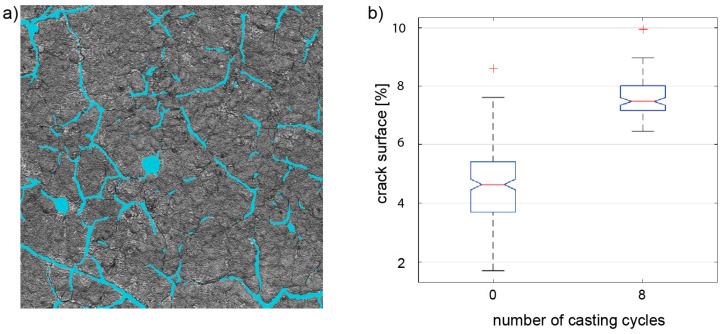
Crack segmentation on AAM-UHPC surface using the HED neural networks: (**a**) detected cracks and (**b**) boxplot of crack surface fraction.

The development of the number of cracks found on the surface of the AAM-UHPC molds was quantified by crack segmentation using a neural network. The results of the analysis of the crack surface are shown in Figure 5. An exemplary image of a laser scanning microscope image of an AAM-UHPC surface with an overlay of the detected cracks in cyan is shown in Figure 5a. This procedure was carried out on 300 images of the surface of the AAM-UHPC molds before and after casting. The percentage of crack pixels of each image was calculated and visualized in the form of boxplots, as Figure 5b depicts. From the boxplots it can be derived that the relative number of cracks rises from 4.6% before to 7.5% after casting. Thus, it can be concluded that the impression obtained from the surface measurements in Figure 4 is valid for the entire mold: In the case of AAM-UHPC, initial cracks grow over the number of casting cycles and lead to failure. Presumably, the crack growth measured is also the cause for the degeneration of the concrete surface over the casting cycles, here a rise in roughness (S_a_) and texture (S_dr_). Additionally, it is assumed that the crack growth is driven by the thermal and mechanical stress applied to the mold. Further research should consider additional crack growth observation, which is essential for the understanding of the cause of failure and a subsequent optimization of the molds to enhance the maximum number of casting cycles.

### 3.3. Concrete and Aluminum Microstructure

#### 3.3.1. EDX-Analysis and Microstructure

**Figure 6:**
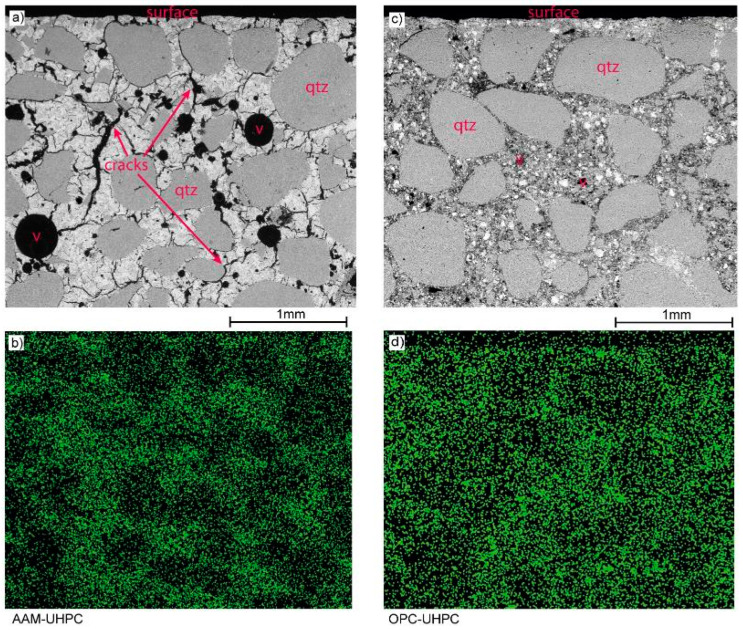
Secondary electron mode images of the cross sections of (**a**) AAM-UHPC and (**c**) OPC-UHPC (qtz—quartz grains, v—air voids). Distribution of aluminum (green) in the microstructure (**b**) AAM-UHPC: (**d**) OPC-UHPC.

After the AAM-UHPC mold could withstand ten casting cycles without failure, it was examined by scanning electron microscopy and energy-dispersive X-ray spectroscopy. A polished section of the AAM-UHPC mold was manufactured and is shown in Figure 6a. The OPC-UHPC mold is also shown in Figure 6b but could only withstand two casting cycles before the surface quality was not suitable for casting any more parts, and the mold failed before a third casting could be conducted. The surface in the upper area of the image has been the aluminum-facing part of the mold.

On the back-scattered electron images, the different microstructures are already apparent: AAM-UHPC exhibits a lot of micro- and macro-cracks (a), whereas OPC-UHPC shows less to no cracks but a very dense microstructure (b), all samples tested had already been in contact with molten aluminum. Therefore, the cause of the cracking cannot be clearly proven. However, it is known that AAM exhibits significantly higher shrinkage values compared to OPC-based concretes and that microcracks were also observed in other investigations without thermal load [24,25]. Therefore, it is assumed that the microcracks are due to shrinkage and macrocracks are attributable to thermal stress. In any case, these microcracks do not appear to be detrimental to thermal stability (Figure 4), and also no aluminum adheres to the surface or impregnates the concrete mold. This has been proven by EDX analyses shown in Figure 6c,d. The regular and fine distribution of the aluminum indicates that it is not introduced by the casting process but already present in the raw material of the UHPCs. The outlines of quartz particles are slightly visible in the aluminum-containing matrix of both materials. Both Portland cement and ground granulated blast-furnace slag, being important components of the UHPC, contain aluminum oxide. Moreover, no gradient of aluminum starting from the surface could be observed to prove impregnation or contamination into the microstructure.

**Figure 7:**
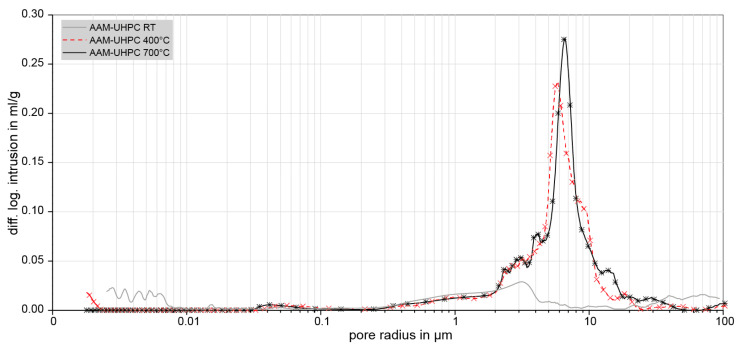
Pore size distributions of AAM-UHPC at room temperature-stored conditions and heat-treated conditions (400 °C and 700 °C).

Respectively, Figure 7 displays the pore size distributions of AAM-UHPC. All samples were stored at room temperature for at least 7 days. The pore size distribution of the untreated light grey-labelled sample was measured, and the red dashed and black labelled samples were heat-treated for the duration of one hour at 400 °C and 700 °C, respectively. After heat treatment, the specimens were allowed to cool to room temperature, and an MIP was performed to investigate the thermal influence on the pore size distribution.

Between 300 and 400 °C, the cellulose fibers degenerate in the binders’ matrix and leave channels of about 10 µm in diameter. The degeneration of the cellulose-based fibers is mainly responsible for the increase in porosity in the 2–20 µm range of the two heat-treated samples. Prior investigations [22,23] had shown similar observations for OPC-UHPC. Generally, the microstructure has been coarsened through the heat treatment. Also, the porosity of the reference sample (RT) was higher than in both the study of Wiemer et al. [23] as well as Wetzel and Middendorf [24], particularly in the range of capillary pores. This could be explained with the slightly different composition of the AAM-UHPC compared to the previous formulations, especially a higher w/b ratio in the concrete-mold composition.

#### 3.3.2. Analysis of Mineral Composition (XRD, TGA)

The phase analyses by XRD and TGA were measured on binder samples without any aggregate to obviate the prominent quartz peaks of the aggregates and focus on the binders’ mineral composition and thermal behavior.

**Figure 8:**
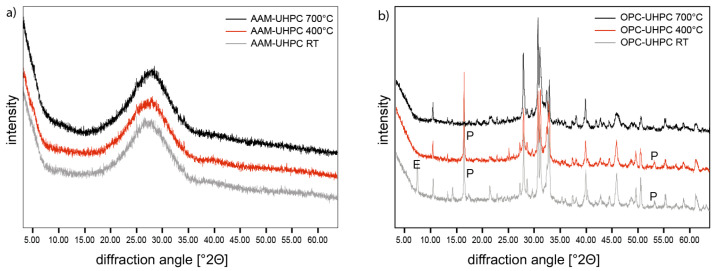
X-ray diffractograms of (**a**) AAM-UHPC and (**b**) OPC-UHPC after storage at room temperature for 7 days (grey), heat-treated at 400 °C for 1 h (red), and at 700 °C for 1 h (black). All samples were manufactured without aggregate.

In XRD (Figure 8), no significant phase transformations or formation of new phases due to thermal loads had been observed in any of the three different treated samples. As expected, ettringite dissipates from OPC-UHPC after the thermal loading. This usually happens at temperatures of 100–200 °C. The ettringite peak is absent in the 400 °C and 700 °C heated samples. Portlandite also decomposes from OPC-UHPC after the heat treatment but at higher temperatures. The Portlandite peaks cannot be found in the 700 °C sample.

The amorphous hump in AAM-UHPC does not shift in position, and its intensity does not diminish significantly. No crystalline phases were detected before or after heat treatment. It is assumed that the phases in AAM are partially N-A-S-(H) phases of nanocrystallinity and therefore X-ray amorphous. This hypothesis is also substantiated by thermogravimetric analyses.

**Figure 9:**
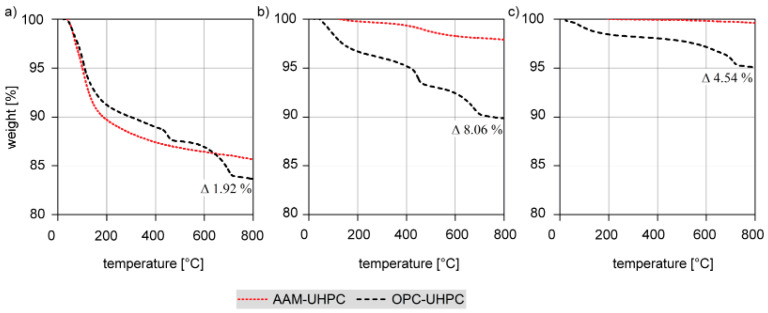
Thermogravimetric analyses of AAM-UHPC (red) and OPC-UHPC (black) after storage at (**a**) room temperature for 7 days, (**b**) heat-treated at 400 °C for 1 h, and (**c**) at 700 °C for 1 h.

TGA measurements show a clear difference in the thermic behavior of AAM-UHPC and OPC-UHPC: When first heated up, OPC-UHPC loses 17% of its weight, mostly due to degradation of the hydration products of OPC: mainly C-S-H phases, portlandite, and ettringite. The weight loss of AAM-UHPC is with 14% a little lower compared to OPC-UHPC (Figure 9a). This may be due to the smaller number of phases containing water. While OPC-UHPC consists of C-S-H phases, which generate the strength of the material and contain chemically bound water, there is less chemically bound water in the polymer-like network of AAM-UHPC [27].

When pre-heated at 400 °C or 700 °C, respectively, the difference is even more significant (Figure 9b,c): AAM-OPC exhibits little to almost no weight loss; the pre-heated OPC-UHPC still loses 10% (pre-heated at 400 °C) and 5% (pre-heated at 700 °C) in weight.

The weight loss of OPC-UHPC increases considerably between 100 °C and 150 °C, at 450 °C, and at 700 °C (Figure 9a,b), which is attributable to the degradation of the main hydration products of Portland cement. C-S-H phases degrade gradually in the range of 100–500 °C and portlandite in the range of 450–550 °C. Moreover, the main binding phases, the C-A-S-H phases, a certain amount of N-A-S-(H) phase contributes to the strength of AAM-UHPC. The latter does not contain chemically bonded water in a hardened state and, therefore, shows an enhanced thermal stability. Nevertheless, the weight loss due to phase degradation is lower for AAM-UHPC than for OPC-UHPC. The AAM-TGA measurements thus prove the suitability of AAM-UHPC for elevated temperature applications.

#### 3.3.3. Cast Aluminum Microstructure

**Figure 10:**
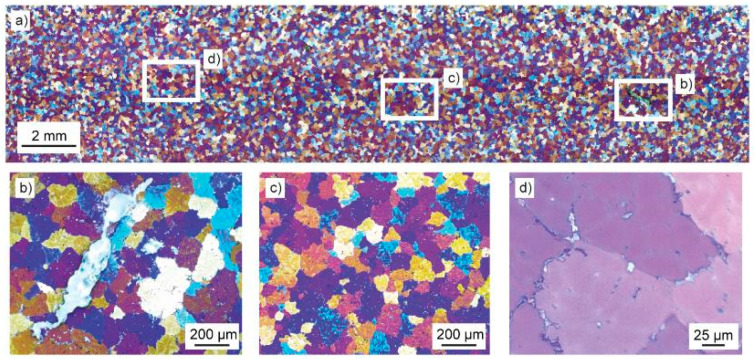
Cross-section of one casted plate (3 mm thick) under polarized light: (**a**) overview, (**b**) macropore, (**c**) microporosity, and (**d**) phases.

One of the main differences between casting in steel and concrete molds lies in the thermal properties of the mold and thus in the cooling of the casted parts. Aiming to investigate whether the concrete formulation tested is feasible to serve as suitable mold material, the microstructure of the casted parts was assessed. Figure 10 shows a cross-section from AlMg4.5Mn0.7 cast in an A-UHPC concrete mold. Due to the etchant applied and the observation under polarized light, grains appear in different colors, depending on their orientation in the probe. As can be seen from the image, the grain distribution appears homogeneous over the specimen, with a slight peak in the grain size in the middle of the plate. In addition, three macroscopic pores/inclusions of irregular shape were observed, as shown in Figure 10a. Moreover, on the microscale, homogeneously distributed pores were found in interdendritic regions as well as at the grain boundaries; see Figure 10b,c. In addition, phases of unknown composition were found in nearly all images analyzed. These phases were primarily located at the grain boundaries and, to a less frequent extent, in the interdendritic regions.

**Figure 11:**
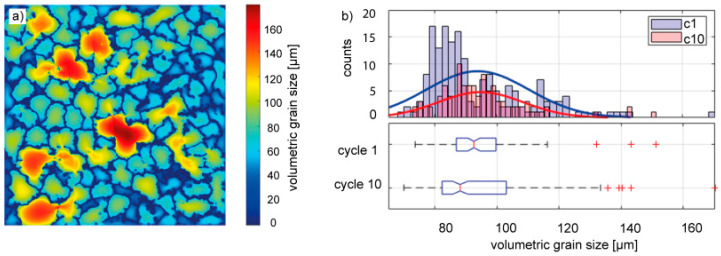
Distribution of the grain sizes measured in both specimens after one casting cycle (c1) and ten casting cycles (c10) conducted with the same pair of AAM-UHPC molds: (**a**) local distribution of grain sizes and (**b**) global distribution of grain sizes (frequency).

Aiming to investigate the size of the grains as well as their distribution in a structured way, an extensive light microscopy analysis was carried out. Hereby the volumetric grain size was determined using the method proposed by Lehto et al. [38] for welded steel joints. Figure 11 shows the result of this analysis by an exemplary light microscopic image with the processed grain size measurement in (a) and a statistical evaluation of the measurements performed on two cross sections taken out of two different aluminum parts cast in AAM-UHPC molds. As it can be seen in Figure 11a, the grain sizes measured were homogeneously distributed along the x- and y-coordinates of the image. Moreover, the grain sizes range between 60 µm and 160 µm. Larger grains seem to be present to a less frequent extent compared to smaller grains. The analysis of all images taken is summarized in Figure 11b. The figure shows that the impressions derived from Figure 10 and Figure 11 are valid for the majority of the 278 images taken from six cutups out of two casting samples. More specifically, the plots confirm that the majority of grain sizes lies in the range between ~75 µm and 130 µm, except for a few outliers. Hereby the outliers are mostly false detections, where bits of the background were detected as grains. Another potential source of error is neighboring grains of low contrast, which were not properly identified as edges by the neural network. Nevertheless, median grain sizes of 88 µm (C1) and 92 µm (C10) were measured over 278 images.

In terms of grain size, grain size distribution, as well as the occurrence of porosity and phases, the results are similar to those achieved by Engler and Miller-Jupp [39]. The authors cast AlMg4.5Mn0.7 using direct chill casting, typically conducted in steel molds, and extensively characterized the outcome in the subsequent study. Furthermore, the authors discovered the composition of the predominant phases, which were Al_6_(Mn,Fe), α-Al(Fe,Mn)Si, and ε-Al_18_(Cr,Mn)_2_Mg_3_. These phases tend to have an impact on the mechanical properties of the casted alloy. Therefore, further research on the correlation of temperature gradient, phase/porosity occurrence, and mechanical properties is recommended.

## 4. Discussion

### 4.1. Concrete Molds

In the course of the investigations carried out, the different properties and the resulting deviating suitability of two UHPC mixtures for use as casting molds were investigated. The differences between OPC-UHPC and AAM-UHPC are already evident in the initial state prior to the first casting cycle. The AAM-UHPC formulation showed shrinkage cracks inside the concrete material (Figure 6) and vast crack networks on the surface (Figure 4) before the first casting cycle. In contrast, the OPC-UHPC molds showed thin microcracks before casting, which significantly increased in width after the first casting cycle. Additionally, the OPC-UHPC sporadically showed spherical pores and pore accumulations (see Figure 4). The surface of the OPC-UHPC molds was smoother compared to AAM-UHPC, resulting in lower S_a_ and S_dr_ values in the LSM measurements presented in Figure 4. Electron microscopic investigations performed showed a continuation of the impression gained on the surfaces: the polished section through the OPC-UHPC mold (Figure 6b) also showed fewer cracks and air voids as well as a generally denser matrix compared to AAM-UHPC.

Discernible phase changes and significantly higher amounts of weight loss, which are due to the degradation of phases containing chemically bonded water within the OPC-UHPC (Figure 9), indicate that the C-S-H phases degenerated during the casting cycles. Therefore, TGA measurements proved the superior thermo-mechanical behavior of AAM-UHPC, especially in pre-heated conditions. The physically and chemically bound water inside the concrete material does not only influence the thermo-mechanical durability; it also seems to have a remarkable impact on the surface quality of the casted part (see Figure 4).

The results of the porosity measurements for AAM-UHPC (Figure 7) confirm the suitability of the material for casting applications. Although the porosity is increased by thermal exposure, this increase is believed to be beneficial for the reduction of water vapor pressure, which would otherwise lead to spalling of the concrete [23]. The increase in porosity due to thermal treatment is mostly located in the capillary pore region and leads to the degeneration of fibers and the growth and extension of microcracks. The differences in the chemical composition of AAM-UHPC and OPC-UHPC could be another possible explanation of the unequal thermal behaviors. Cement pastes containing ground blast slag show superior thermal stability compared to OPC because of the lower portlandite content [40]. Portlandite degenerates above 400 °C and partly recrystallizes again according to an existing ambient humidity. The evaporation of the C-S-H interlayer water is reversible, too, and emerges in OPC-UHPC but not in AAM-UHPC (see differences in weight loss in Figure 9). Thus, the lower water content in AAM-UHPC compared to the OPC-UHPC could explain the superior thermal stability.

The AAM-UHPC formulation could be further adapted to the extent that a shift from C-A-S-H to N-A-S-(H) phases could result in increased thermal stability. This premise is supported by the previously described investigations and the lower water content in the N-A-S-(H) phases. The selection of low-calcium precursors would shift the ratio towards N-A-S-(H) phases. For example, the slag could be partially replaced by metakaolin, which is rich in aluminum and low in calcium. However, the use of metakaolin leads to poorer workability and an increased water requirement compared to GGBFS. Whether replacing GGBFS with metakaolin will result in a processable mixture that is also thermally stable remains to be tested.

However, the OPC-UHPC mold (with fewer initial defects) failed after two casting cycles, whereas the AAM-UHPC mold (with more initial defects) remained operational for at least ten cycles. This observation leads to the presumption that the initial cracks present in both UHPC formulations contribute to the mold failure, but in a different way than intuitively assumed. In general, both UHPC formulations contain water, with OPC containing more than AAM-UHPC, which vaporizes when the aluminum melt comes into contact with the concrete mold. The interaction between the vaporized water and concrete, as well as aluminum, has an impact on mold and casting. In the case of AAM-UHPC, the initial crack networks and the overall rough and strongly textured surfaces (S_dr_), compared to OPC-UHPC, support the distribution of vapor pressure across the entire surface area of the mold. This enables an equal distribution of vapor pressure and consequently a decrease in specific thermal and mechanical load. Additionally, it is assumed that the vapor pressure in the AAM molds is lower due to the reduced water content. This hypothesis is supported by the documented degeneration of the surfaces over the casting cycles, evidenced by an increase in mold roughness (S_a_). For OPC-UHPC, it is presumed that the smooth (S_a_) and untextured surface (S_dr_) work unfavorably for the distribution of vapor pressure. It is assumed that hot steam is pressed into cavities and microcracks and therefore not equally distributed over the mold surface. Consequently, the specific load increases in the region of defects, causing microcracks to grow in length and width. The same hypothesis is assumed to be valid for the distribution of thermal loading in both cases.

Consequently, the specific mechanical and thermal loading resulting from the casting process is lower for each infinitesimal volume element on the AAM mold surface compared to OPC. Apart from the thermal and mechanical loading caused by vapor pressure, the molds are subjected to differing mechanical stresses from the test setup, which are not linked to the formulation of the UHPC and promote crack growth. Firstly, clamping forces resulting from the positioning of the molds in the casting machine by the use of a clamping device impose additional stress on the UHPC materials. Secondly, tolerances in the flatness of the molds contact surfaces cause additional local stress when the molds are clamped together. Furthermore, OPC-UHPC formulations are known to be more brittle compared to AAM-UHPC. This lack of ductility consequently leads to an increase in crack growth per casting cycle in OPC molds. As a result, OPC-UHPC molds, which are more susceptible to exhibiting large crack growth rates, experience higher stress during the casting process due to their surface appearance and last fewer cycles compared to AAM-UHPC molds. Nevertheless, both formulations resist the thermal and mechanical stresses resulting from the casting process and are thus considered to be hot shock-resistant to a certain extent. Although a plate was cast, resulting in a planar state of stress, the UHPC materials showed resistance to more complex, presumably multiaxial, notch stresses in the corners and near the edges of the mold.

### 4.2. Aluminum Casting

Regarding the aluminum castings, it was reported that the texture of the concrete surface as well as its roughness were not depicted in the aluminum casting. This result of the confocal laser microscopic investigations is presumably a consequence of the wetting behavior of the concrete. It is assumed that the surface energy of the aluminum melt is higher than the critical surface energy of the concrete. The aluminum is therefore not completely wetting the concrete surface and consequently not transferring the mold surface texture and roughness to the aluminum casting. In addition, the grain sizes and the distribution of the grains detected by metallographic examination and subsequent light microscopic analysis were comparable to those reported by Engler and Miller-Jupp [39]. The authors applied the same alloy but cast the aluminum in steel molds. The microstructure analysis of the current study also reveals that a sound casting quality regarding porosity, inclusions, and other defects was achieved. Therefore, AAM-UHPC is considered a feasible mold material for aluminum castings, which leads to the same microstructure compared to state-of-the-art castings in steel molds. The pore solution that escapes from the concrete molding materials during the casting process is alkaline. The extent to which the alkalinity of the pore solution influences the surface and microstructure of the aluminum should also be considered in the future. In general, the pore solution of AAM is more alkaline than that of OPC, but since the aluminum parts have a higher surface quality, it can be assumed that the amount of fluid, which is larger in OPC-UHPC (Figure 9), has a stronger influence than the alkalinity.

### 4.3. Aluminum Castings with Concrete Mold

Concerning the process of aluminum casting using molds made from UHPC, the current study yielded promising results but also revealed the limitations of the proposed process. While aluminum castings in concrete molds exhibited a surface and microstructural appearance comparable to those manufactured in steel molds, the durability of the tested molds was restricted to two and ten casting cycles for OPC-UHPC and AAM-UHPC molds, respectively. Earlier discussions on potential causes of mold failure highlighted the significant influence of both the experimental setup and the UHPC formulation on the maximum number of casting cycles. Therefore, with the selection of the appropriate formulation and a reduction in the initial loading of the concrete mold, the proposed process has the potential to bridge the gap between sand and permanent mold casting in terms of mold durability and manufacturing efficiency. Casting in UHPC molds could replace multiple sand-casting molds with a single, more durable mold or enable the economical production of larger parts that are impractical to produce in steel molds. Additionally, the study suggests exploring the possibility of casting thin-walled or small parts in concrete molds, although there may be limitations in mold accuracy due to the texture of the concrete not being transferred to the casting. Furthermore, the study found that AAM-UHPC appears resistant to non-planar stress states, such as those occurring at edges, corners, and notches. To better understand concrete molds’ robustness against multiaxial fatigue induced by shrinkage forces, especially when casting complex geometries, further research is warranted. Concrete’s multiaxial stress induced by shrinkage forces is further compounded by the strong gradient in thermal expansion coefficients between aluminum and concrete. Planned research endeavors aim to address these challenges and enhance mold durability, with a target of achieving 50–100 casting cycles.

## 5. Conclusions and Outlook

### 5.1. Conclusions

The study explores the feasibility of using Ultra High Performance Concrete (UHPC) as a mold material for aluminum castings. Two concrete formulations, AAM-UHPC and OPC-UHPC, were tested for mold utility, using PTFE molds to create UHPC casting molds. Molds were employed for pressure-assisted mold casting of AlMg4.5Mn0.7. The results can be concluded to the following key points:

- The application of the molds in low-pressure casting achieved over 99% accuracy in geometry compared to CAD models.
- OPC-UHPC molds lasted at least two casting cycles, while AAM-UHPC molds lasted at least ten cycles. Surface quality degraded over cycles, with AAM-UHPC showing rougher surfaces, which did not transfer to the aluminum casting.
- The failure of the molds is driven by a combination of thermo-mechanical stress from the casting process and additional mechanical stress from the test setup.
- UHPC formulation, particularly water content in OPC-UHPC, is crucial for mold durability; cellulose fibers enhance thermo-mechanical resistance.
- Castings displayed homogeneously distributed grains (~90 µm) with low porosity and few macroscopic inclusions. The amount and appearance of defects can be compared with aluminum castings performed using the same alloy.
- AAM-UHPC, in particular, showed good thermomechanical resistance to the basic conditions of the casting process, probably owing to a different phase composition of the binder matrix with less chemically bound water.

The proposed process chain could potentially bridge the gap between lost foam casting (sand/investment) and gravity die casting.

### 5.2. Outlook and Limitations of This Study

The findings confirm the general feasibility of UHPC as a mold material for semi-permanent mold casting of aluminum and present important core aspects of the interaction between concrete and casting, but further boundary conditions remain unclear. For example, the influence of the concrete mold material on the resulting mechanical properties of the casting requires further investigations. The interaction between the UHPC mold and the aluminum melt has to be investigated in more detail to understand the root causes of the concrete surface’s degradation over multiple casting cycles. Acquiring fundamental knowledge about these interaction processes promises to significantly enhance the durability of UHPC molds, which was identified as a major drawback of the tested concrete molds. Future research should also consider the molds’ resistance to multiaxial stress states resulting from shrinkage forces. Enhancing mold resistance to multiaxial stress is essential for casting more complex geometries and larger parts. Additionally, the design of the experimental setup, aimed at reducing the initial loading of the UHPC molds, should be addressed in subsequent studies in this area.

During the casting process, water vapor and so-called pore solution escape from the concrete molding materials. The pore solution consists of different ions (mostly Ca, Na, K) dissolved in water and is alkaline. The extent to which the alkalinity of the pore solution influences the surface and microstructure of the aluminum should also be considered in the future. In general, the pore solution of AAM is more alkaline than that of OPC, but since the aluminum parts have a higher surface quality, it can be assumed that the amount of evaporated fluid, which is larger in OPC-UHPC (Figure 9), has a stronger influence than the chemical composition of the pore solution. Even though the importance of the water content and the temperature influence became clear, further investigations are required to gain a better understanding of the basic effect mechanisms. The reason for the different wetting behavior of AAM-UHPC and OPC-UHPC also represents a key aspect of further investigations. The influence of quartz inversion occurring at 573 °C in the aggregates of the concretes also needs to be investigated in terms of its effect on the thermal stability of the molds. It is assumed that quartz-free aggregates could provide better thermal stability of the molds.

## 6. Patents

The disclosure document for the patent “Casting mold for casting metals” (DE 10 2022 131 971 A1) was published in 2024.

## Figures and Tables

**Table 1:** Masses of AAM-UHPC and OPC-UHPC (in kg/m^3^) for a mixing volume of 1 L.

Component	AAM-UHPC	OPC-UHPC
Potassium Waterglass	392	-
Water	-	230
GGBFS	474	-
Portland Cement	-	718
Silica Fume	292	152
Hydrophobic Agent	16	14
Superplasticizer (PCE)	-	21
Quartz Powder	156	201
Quartz Sand	975	986
RC Fibers	3	3

## Data Availability

The original contributions presented in this study are included in the article. Further inquiries can be directed to the corresponding author.
